# Impact of Phosphate Variability in Patients Undergoing Hemodialysis

**DOI:** 10.3390/nu17091528

**Published:** 2025-04-30

**Authors:** Seok-Hui Kang, So-Young Park, Yu-Jeong Lim, Bo-Yeon Kim, Ji-Young Choi, Jun-Young Do, A-Young Kim

**Affiliations:** 1 Division of Nephrology, Department of Internal Medicine, College of Medicine, Yeungnam University, Daegu 42415, Republic of Korea; kangkang@ynu.ac.kr (S.-H.K.);; 2 Department of Physiology, College of Medicine, Yeungnam University, Daegu 42415, Republic of Korea; 3 Healthcare Review and Assessment Committee, Health Insurance Review and Assessment Service, Wonju 26465, Republic of Korea

**Keywords:** hemodialysis, phosphate, dementia, survival

## Abstract

Background: There are few studies investigating the relationship between phosphate level variability and clinical prognosis in hemodialysis (HD) patients. This study aimed to evaluate the impact of phosphate variability on clinical outcomes in maintenance HD patients using a population-based cohort. Methods: We analyzed data from 55,225 patients who underwent periodic HD quality assessments and claims review. Phosphate variability was assessed using the residual standard deviation (SD) from a within-subject linear regression model based on six phosphate measurements per patient. Participants were categorized into quartiles based on phosphate variability. A balanced cohort was created using generalized boosted models for relevant covariates. Results: After weighting, the residual phosphate SDs were 0.35 ± 0.00 mg/dL (Q1), 0.57 ± 0.00 mg/dL (Q2), 0.79 ± 0.00 mg/dL (Q3), and 1.22 ± 0.00 mg/dL (Q4). The mean follow-up duration across all quartiles was 50 ± 0.2 months. Multivariable Cox regression analysis revealed that patients in the Q4 group had a significantly higher hazard ratio (HR) for all-cause mortality and dementia compared with the Q1 group. Among all quartiles, Q1 showed the lowest HR for dementia. These trends were consistent with those observed in spline curve analyses. However, no significant association was found between phosphate variability and cardiovascular events. Conclusions: High phosphate variability was associated with increased risk of all-cause mortality and dementia in maintenance HD patients.

## 1. Introduction

Chronic kidney disease (CKD) has recently gained attention as a chronic disease with rapidly increasing healthcare expenditures [1]. CKD commonly progresses to end-stage kidney disease, at which point patients require either transplantation or dialysis. Among these treatment modalities, hemodialysis (HD) is the most widely performed. HD patients have a significantly higher mortality rate compared to the general population [2]. Therefore, identifying appropriate risk factors and effectively managing them is crucial for improving survival in these patients. Phosphate excretion is markedly impaired in HD patients compared to those with normal renal function. In addition, due to its inherent properties, phosphate is not efficiently removed through dialysis, contributing to hyperphosphatemia [3]. Hyperphosphatemia plays a key role in chronic kidney disease–mineral and bone disorder (CKD–MBD), leading to complications such as bone abnormalities and vascular calcification, which in turn contribute to various adverse outcomes [4].

The KDIGO guidelines recently recommended maintaining phosphate levels toward normal range [4]. However, phosphate is an essential nutrient associated with favorable nutrition, and studies have suggested a reverse epidemiology phenomenon, where lower phosphate levels might be indicative of poorer nutritional status [5]. Furthermore, phosphate levels are influenced by multiple factors, including the use of phosphate binders (PPBs), residual renal function, and intact-parathyroid hormone (iPTH) levels, highlighting the need to consider additional related factors beyond a single phosphate measurement.

Changes in serum phosphate levels are associated with adverse clinical outcomes. Hypophosphatemia can reduce cellular ATP production, leading to general weakness and, in severe cases, myopathy or neurological complications [6]. Hyperphosphatemia is linked to secondary hyperparathyroidism and vascular calcification, which contribute to increased morbidity and mortality in patients [7]. However, phosphate variability shares similarities with these conditions but also presents distinct features. Given that fluctuations in various clinical and laboratory parameters—such as systolic blood pressure, ultrafiltration volume (UFV), and hemoglobin—have been reported to affect prognosis in HD patients, it is plausible that phosphate level variability may also impact clinical outcomes [8,9,10]. If variability in serum phosphate levels is considered a reflection of impaired physiological homeostasis, potential contributors may include inadequate feedback regulation and external perturbations such as dietary inconsistency, poor medication adherence, or dialysis-related factors. Therefore, phosphate variability may serve as an additional indicator beyond a single measurement of serum phosphate level. Phosphate variability tends to be more pronounced in conditions where the bone fails to act as an adequate buffer, such as in adynamic bone disease, and Wang et al. [11] showed a positive association between phosphate variability and coronary vascular calcification. Thus, it may ultimately be linked—either directly or indirectly—to adverse clinical outcomes [11,12]. However, there are few studies investigating the relationship between phosphate level variability and clinical prognosis in HD patients. Therefore, this study aims to evaluate the effect of phosphate level variability on clinical outcomes in maintenance HD patients using a population-based cohort.

## 2. Materials and Methods

### 2.1. Data and Population

In our retrospective study, datasets from patients who underwent periodic HD quality assessments and their claims data were analyzed [13,14]. Briefly, the first HD quality assessment program was performed between October and December 2010. Data from the sixth (March and August 2018) and seventh (October and March 2021) HD quality assessment programs were used in this study. These programs included adult patients (≥18 years) who had undergone maintenance HD (≥3 months and ≥2 times per week). Data from the relevant HD quality assessment and claims data for all patients were analyzed.

Among 69,967 patients included in the sixth and seventh assessments, we excluded the following groups: repeat participants (*n* = 17,269), patients undergoing HD through a catheter (*n* = 900), those with insufficient data (*n* = 181), those with at least one missing phosphate measurement during the assessment (*n* = 288), and those identified as outliers within the 0.2% range on six measurements (*n* = 1104). Overall, 50,225 patients were included in this study. This study was approved by the institutional review board of Yeungnam University Medical Center (approval no. YUMC 2023-12-012). Informed consent was not required, as patient records and information were anonymized and de-identified before analysis. The institutional review board of the Yeungnam University Medical Center waived the informed consent requirement owing to the retrospective nature of the study. This research was conducted in adherence to ethical standards outlined in the Declaration of Helsinki.

### 2.2. Study Variables

During each HD quality assessment, data were collected on age, sex, HD vintage (months), and vascular access type. Additional clinical measures included hemoglobin (g/dL), body mass index (kg/m^2^), Kt/V_urea_, serum albumin (g/dL), serum calcium (mg/dL), serum phosphate (mg/dL), serum creatinine (mg/dL), and UFV (L/session). These data were collected monthly, with all laboratory values averaged from the monthly recordings. Kt/V_urea_ was calculated using the Daugirdas equation [15]. Phosphate measurement was also collected monthly, as a value of Monday or Tuesday among a month. Mean phosphate value was presented as average value of 6 values over a 6-month period. All laboratory findings, including serum phosphate, were measured immediately before the dialysis session, regardless of food intake. The phosphate variability was calculated using the residual standard deviation (SD) derived from a within-subject linear regression model with six phosphate values for each patient [8]. Participants were divided into the four quartile groups based on the phosphate variability: first quartile (Q1), second quartile (Q2), third quartile (Q3), and fourth quartile (Q4).

Table S1 presents the medication codes. Medications, including renin–angiotensin system blocker (RASB), aspirin, clopidogrel, statins, anti-hypertensive drugs, PPB, vitamin D agents (including paricalcitol), cinacalcet, and calcium supplement (calcium gluconate, calcium citrate, calcium lactate, or calcium phosphate) were evaluated. Medication use was defined as ≥1 prescription identified during the HD quality assessment program. The use of PPB was divided into calcium-containing (calcium acetate or calcium carbonate) phosphate binders (CPPBs) or non-calcium-containing (sevelamer or lanthanum) phosphate binders (NCPPBs). If the patients used two PPBs, the type was defined as most frequently used medication. Comorbidities were assessed for 1 year before the HD quality assessment. The Charlson comorbidity index (CCI) was used to define comorbidities, which includes 17 comorbidities. CCI scores were calculated for all patients [16,17]. Additionally, myocardial infarction (MI) or congestive heart failure (CHF) and atrial fibrillation (Afib) were identified using ICD-10 codes.

Outcomes were evaluated from the end point of each HD quality assessment program to the follow-up end point (June 2024). All-cause mortality, cardiovascular events (CVE), and dementia were analyzed. Data on patient death were obtained from the Health Insurance Review and Assessment Service, and patients who changed to peritoneal dialysis or received kidney transplantation without experiencing an event were censored at the time of transfer. The incidence of CVE including MI, stroke, and revascularization regardless of survival or death, was evaluated as previously described [18]. The incidence of dementia was evaluated using ICD-10 codes. The incidence of these events was evaluated using patients without these events for 6 months during the HD quality assessment program and 1 year before the program. Comorbidities and clinical conditions were identified using the following ICD-10 codes: MI (I21, I22, I252); CHF (I43, I50, I099, I110, I130, I132, I255, I420, I425–I429, P290); peripheral vascular disease (I70, I71, I731, I738, I739, I771, I790, I792, K551, K558, K559, Z958, Z959); cerebrovascular disease (G45, G46, I60–I69, H340); dementia (F00–F03, G30, F051, G311); chronic pulmonary disease (J40–J47, J60–J67, J278–J279, J701, J703, J684); rheumatologic disease (M05–M06, M32–M34, M315, M351, M353, M360); peptic ulcer disease (K25–K28); mild liver disease (B18, K73, K74, K700–K703, K709, K713–K715, K717, K760, K762–K764, K768, K769, Z944); diabetes without complications (E100–E101, E106, E108–E111, E116, E118–E121, E126, E128–E131, E136, E138–E141, E146, E148, E149); diabetes with complications (E102–E105, E107, E112–E115, E117, E122–E125, E127, E132–E135, E137, E142–E145, E147); hemiplegia/paraplegia (G81, G82, G041, G114, G800, G830–G834, G839); any malignancy (C00–C26, C30–C34, C37–C41, C43, C45–C58, C60–C66, C81–C88, C90–C97); moderate to severe liver disease (I850, I859, I864, I982, K704, K711, K721, K729, K765–K767); metastatic tumor (C77–C80); and AIDS/HIV (B20–B22, B24).

### 2.3. Statistical Analyses

Data were analyzed using SAS Enterprise Guide v.7.1 and R v.3.5.1. Categorical variables were presented as frequencies and percentages, while continuous variables were expressed as means with SDs. Statistical significant differences between categorical variables were assessed using Pearson’s χ^2^ test or Fisher’s exact test. Differences between continuous variables were examined using a one-way analysis of variance with Tukey’s post hoc test.

There were significant differences in baseline characteristics among the four groups. We used the inverse probability of treatment weighting to balance these characteristics and ensure that the results of our analyses were not biased. We created the balanced cohort for the 4 groups using generalized boosted models for the following variables: age; sex; body mass index; the presence of diabetes; types of vascular access; CCI score; HD vintage; UFV; Kt/V_urea_; hemoglobin, albumin, creatinine, phosphate, and calcium levels; the administration of aspirin, statin, RASB, anti-hypertensive drugs, clopidogrel, PPB, vitamin D agents, cinacalcet, or calcium supplements; and the presence of MI or CHF or Afib. Propensity scores were used to calculate the inverse probability treatment weights. Finally, we defined the balanced cohort as a sample with weights assigned to each case and continuous variables were presented as means and standard errors. *p*-values were tested using a general linear model using a complex survey design, including sample weights.

Survival estimates were calculated using Cox regression analyses and presented as hazard ratios (HRs) and confidence intervals. Multivariable Cox regression analyses were adjusted for the following variables: age, sex, body mass index, vascular access type, diabetes, HD vintage, CCI score, UFV, Kt/V_urea_, hemoglobin, serum albumin, serum creatinine, serum phosphate, and serum calcium levels; use of RASB, anti-hypertensive drug, statins, clopidogrel, aspirin, PPB, vitamin D agents, cinacalcet, or calcium supplements; presence of MI or CHF; and presence of Afib. All variables were entered into the models simultaneously. Subgroup analyses were based on age, sex, median HD vintage, median CCI score, mean phosphate level, and use of PPB. A restricted cubic spline curve was used to evaluate non-linear relationships between the phosphate variability and patient death or incidence of CVE or dementia, which was adjusted for covariates. Statistical significance was determined at *p* < 0.05.

## 3. Results

### 3.1. Baseline Characteristics

Variables associated with phosphate are summarized in Table 1.

The residual SDs of the phosphate levels before weighting were 0.34 ± 0.09 mg/dL (Q1), 0.57 ± 0.06 mg/dL (Q2), 0.80 ± 0.07 mg/dL (Q3), and 1.25 ± 0.29 mg/dL (Q4). After applying weighting, the residual SDs were 0.35 ± 0.00 mg/dL (Q1), 0.57 ± 0.00 mg/dL (Q2), 0.79 ± 0.00 mg/dL (Q3), and 1.22 ± 0.00 mg/dL (Q4).

The unweighted cohort comprised 12,557 patients in Q1, 12,556 in Q2, 12,556 in Q3, and 12,556 in Q4 (Table S2). Patients in Q4 tended to be younger and had higher UFVs, serum albumin, mean phosphate, and serum creatinine levels. This group also had higher proportions of patients using RASBs, antihypertensive drugs, NCPPBs, vitamin D agents, and cinacalcet. In contrast, Q4 had lower proportions of male patients, individuals with diabetes, and those with Afib.

To account for these baseline differences, an inverse probability of treatment weighting was applied, and a substantial overlap in propensity scores was observed (Figure S1). After weighting, the respective quartiles included 48,817 (Q1), 49,310 (Q2), 49,158 (Q3), and 47,730 (Q4) patients (Table 2).

The weighting process effectively minimized imbalances in baseline characteristics across the groups.

### 3.2. Patient Status as Stratified by Phosphate Variability

The mean follow-up duration in the weighted cohort was 50 ± 0.2 months for all quartiles. At the end of the follow-up period, the distribution of patients across the outcomes of survival, death, transfer to peritoneal dialysis, and kidney transplantation was as follows: Q1: 31,198 (63.9%) survived, 14,795 (30.7%) died, 93 (0.2%) transferred to peritoneal dialysis, and 2551 (5.2%) received a kidney transplant; Q2: 31,579 (64.0%) survived, 15,245 (30.9%) died, 76 (0.2%) transferred to peritoneal dialysis, and 2410 (4.9%) received a kidney transplant; Q3: 31,444 (64.0%) survived, 15,357 (31.2%) died, 74 (0.2%) transferred to peritoneal dialysis, and 2283 (4.6%) received a kidney transplant; and Q4: 30,000 (62.9%) survived, 15,294 (32.0%) died, 75 (0.2%) transferred to peritoneal dialysis, and 2361 (4.9%) received a kidney transplant (*p* < 0.001). The proportion of deaths at the end of follow-up was highest in the Q4 group. The 5-year patient survival rates were 68.0%, 67.4%, 67.3%, and 66.7% in the Q1, Q2, Q3, and Q4, respectively. The 5-year CVE-free rates were 79.2%, 80.0%, 79.2%, and 78.9% in the Q1, Q2, Q3, and Q4, respectively. The 5-year dementia-free rates were 91.5%, 91.4%, 91.1%, and 90.3% in the Q1, Q2, Q3, and Q4, respectively.

### 3.3. Group-Specific Survival Analyses

We also examined the relationship between phosphate variability and the outcomes of patient survival, CVEs, and dementia. Multivariable Cox regression analysis showed that patients in the Q4 group had a greater HR for all-cause mortality and dementia compared to the Q1 group (Table 3).

**Figure 1:**
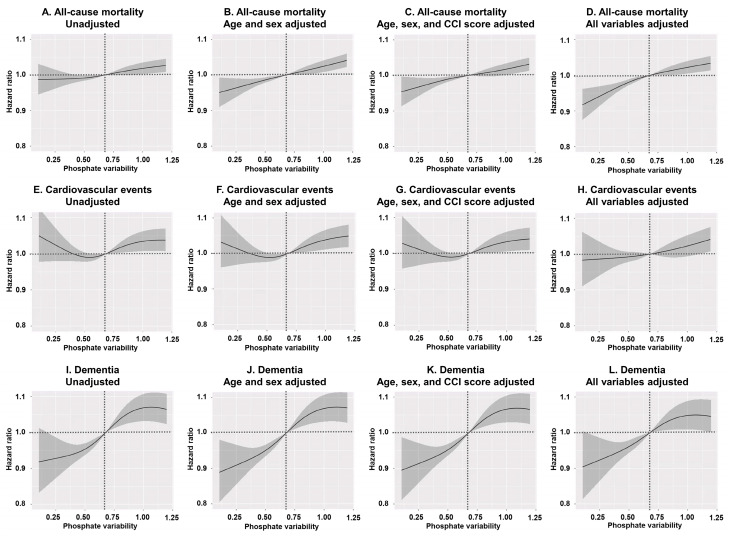
Spline curves illustrating the association between phosphate variability and clinical outcomes (hazard ratio and 95% confidence interval): (**A**–**D**) all-cause mortality, (**E**–**H**) cardiovascular events, and (**I**–**L**) dementia. (**A**,**E**,**I**) show unadjusted models. (**B**,**F**,**J**) are adjusted for age and sex. (**C**,**G**,**K**) are adjusted for age, sex, and CCI score. (**D**,**H**,**L**) are fully adjusted for the following variables: age; sex; body mass index; vascular access type; diabetes status; hemodialysis vintage; CCI score; ultrafiltration volume; Kt/V_urea_; hemoglobin; serum albumin; serum creatinine; mean phosphate and serum calcium levels; use of renin-angiotensin system blockers, statins, clopidogrel, aspirin, antihypertensive medications, vitamin D analogs, phosphate binders, calcium supplements, and cinacalcet; and the presence of myocardial infarction, congestive heart failure, or atrial fibrillation. Dot line reveal 1.0 value of hazard ratio for patients with 0.68 mg/dL of phosphate variability. Abbreviations: CCI, Charlson Comorbidity Index.

The Q1 group had the lowest HR for dementia among the four groups. These results are consistent with the trends observed in the spline curves (Figure 1).

The spline curves, derived from the multivariable model, also demonstrate that increasing phosphate variability (relative to the median of 0.68 mg/dL) is associated with an increased risk of all-cause mortality and dementia. However, no association was found between CVE and phosphate variability groups. No significant differences were observed across the various adjusted models.

### 3.4. Subgroup Analyses

To investigate the influence of specific patient characteristics on the relationship between phosphate variability and outcomes, we conducted subgroup analyses. These analyses were stratified by sex, age (<65 or ≥65 years), HD vintage (<41 or ≥41 months), CCI score (<8 or ≥8), PPB use (CPPB, NCPPB, or no PPB), and mean phosphate levels (<3.5, 3.5–5.5, or >5.5 mg/dL). The lower HR observed for all-cause mortality in the Q1 group was generally consistent across most subgroups (Table S3). For the outcome of CVEs, statistical significance was weak in the majority of subgroups (Table S4). A prominent protective effect of the Q1 group against dementia was observed in subgroups characterized by low CCI, female, younger age, short HD vintage, or PPB use (Table S5). Interestingly, in patients with phosphate level below 3.5 mg/dL, the Q4 group had a lower risk of both all-cause mortality and dementia compared to the Q1 group. However, in patients with phosphate above 3.5 mg/dL, the Q4 group showed a trend toward increased risk for both outcomes. In addition, we plotted spline curves between phosphate variability and clinical outcomes using subgroups based on age or sex (Figure S2). The trends observed in these spline curves were consistent with those found in the subgroup analyses of the multivariable Cox regression models.

## 4. Discussion

This study analyzed data from 50,225 patients undergoing HD who participated in the nationwide HD quality assessment program. Phosphate variability was calculated from 6 months of monthly phosphate measurements and categorized into quartiles. To account for differences in baseline characteristics, we used inverse probability of treatment weighting to create a balanced cohort. Our results demonstrated that patients with high phosphate variability experienced greater all-cause mortality and dementia compared to those with low variability. The lower HR for all-cause mortality in the Q1 group was observed across most subgroups. For the outcome of dementia, a prominent protective effect of the Q1 group was evident in subgroups with low CCI, female sex, younger age, short HD vintage, or PPB use. Furthermore, we found that the relationship between phosphate variability and clinical outcomes varied depending on the patient’s phosphate levels.

Phosphate levels are known to vary significantly between HD sessions, often requiring substantial modifications to treatment [19]. This variability may impact patient prognosis through several pathways: direct changes in serum phosphate levels, indirect effects of phosphate-related factors such as malnutrition, or associated biochemical alterations. Although research exploring the direct association and pathogenesis of phosphate variability is limited, some clinical studies have used variability indicators to assess its impact on all-cause mortality. Ter Meulen et al. used directional range as a measure of variability and showed that higher variability was associated with all-cause mortality [20]. Similarly, Zhu et al. studied maintenance HD patients and found that higher variability indicators, such as the coefficient of variation and SD, were associated with increased mortality [21]. Our study’s findings are consistent with these previous reports. While we observed a statistically significant association between phosphate variability and all-cause mortality (an outcome influenced by various conditions, including CVEs and infections), the association with CVE alone was not statistically significant in most cohorts. This suggests that phosphate variability may have an independent impact on other outcomes, beyond its influence on CVEs. The association between phosphate variability and all-cause mortality was modest, and the results from the weighted cohort were similar with those from most subgroups.

A key finding of our study is the association between phosphate variability and dementia, with greater variability associated with increased risk. Prior research has established a link between phosphate levels and cognitive function, demonstrating that low and high phosphate levels can negatively impact cognition by altering ATP production and the fibroblast growth factor 23 (FGF23) [6,22,23]. These previous findings provide a biological rationale for our observed association between phosphate variability and dementia. Our study suggests that maintaining stable phosphate levels may be important for minimizing dementia risk. Subgroup analyses revealed a particularly strong association between phosphate variability and dementia in subgroups with relatively favorable prognoses, including those with low CCI, female sex, younger age, short HD vintage, and PPB user. In contrast, established risk factors for dementia, such as severe disease and multiple comorbidities, likely acted as confounders, attenuating the observed effect of phosphate variability in these higher-risk groups. Interestingly, in the subgroup with low serum phosphate levels, increased phosphate variability was paradoxically associated with reduced dementia risk, suggesting that fluctuating between low and normal phosphate levels may be preferable to consistently maintaining low levels.

The residual SD, SD, coefficient of variation, maximum–minimum difference, or average real variability have been widely used to assess variability [24]. Although average real variability and the maximum–minimum difference are easy to calculate, they are susceptible to the influence of outliers and do not incorporate the SD. The coefficient of variation (SD/mean) is unit-independent, but this measure is highly sensitive to mean values. Residual SD adjusts for time trends and captures pure intra-individual variability, with minimal influence from mean level changes. However, it assumes a linear trajectory and requires model-based estimation, which may limit its use in non-linear settings. Although there is no consensus on a gold-standard approach method for evaluating variability, residual SD may serve as an ideal approach to capture true variability, despite its computational complexity. Therefore, we used residual SD to assess variability in this study.

Our study has several limitations that should be acknowledged. First, the retrospective nature of the study and the significant differences in baseline characteristics among the four groups, while addressed using inverse probability of treatment weighting, multivariable analyses, and subgroup analyses, could not be completely mitigated. Although this large-scale study employed inverse probability of treatment weighting and multivariable adjustments to mitigate confounding, residual unmeasured confounders cannot be entirely excluded. A more robust design—such as single-center or multicenter study with access to detailed clinical and laboratory data—would better ensure comprehensive adjustment. Ultimately, a randomized controlled trials are required to eliminate hidden confounders and establish causality. Therefore, our findings should be interpreted as supportive evidence that provides a rationale for future confirmatory studies. Second, phosphate variability was assessed over a limited period of 6 months. Additionally, data for medications and dementia, which were derived from ICD-10 codes or claims data. The use of insurance claims data and ICD-10 codes to define medication use, dementia diagnosis, and comorbidities may have introduced under-reporting or misclassification. Although these codes are standardized and routinely used for administrative and reimbursement purposes, they may not fully capture the clinical subtleties or disease severity. This may have affected the accuracy of certain variables in our analysis. Third, several important indicators related to phosphate levels and patient prognosis were not available for analysis. Specifically, we lacked data on key markers of CKD–MBD, such as iPTH, fibroblast growth factor 23 (FGF23), vascular calcification, and alkaline phosphatase, which limited our ability to isolate the independent effect of phosphate variability. This study utilized a nationwide dataset obtained as part of a quality assessment program for all HD centers in South Korea, rather than data collected specifically for research. To ensure cost-effectiveness and broad applicability, the dataset focused on parameters with strong clinical evidence that were accessible across most HD centers. Consequently, several important CKD–MBD-related variables parameters, such as iPTH, FGF23, alkaline phosphatase, vascular calcification scores, and detailed nutritional assessments, were not included. Their absence may have limited our ability to adjust for potential confounders and to explore the independent or interactive effects of phosphate variability more comprehensively. Nevertheless, we attempted partial adjustment using available laboratory data and by considering South Korea’s standardized reimbursement policies, which are applied uniformly across HD centers. For example, our dataset includes key CKD–MBD indicators such as serum calcium and phosphate. In South Korea, non-calcium-based phosphate binders are reimbursed only for patients with serum phosphate ≥ 5.5 mg/dL and a calcium–phosphate product ≥ 55 mg^2^/dL^2^, which indirectly reflects the presence of hyperphosphatemia and vascular calcification risk. Similarly, the prescription of calcium supplements may suggest hypocalcemia, even when not used as phosphate binders. Active vitamin D analogs are also reimbursed based on iPTH levels—calcitriol for iPTH ≥ 200 pg/mL, and paricalcitol or cinacalcet for iPTH ≥ 300 pg/mL—indicating probable secondary hyperparathyroidism. Additionally, although imperfect, nutritional status was approximated using body mass index, serum albumin, and creatinine, which are traditionally employed as surrogate markers. We acknowledge, however, that these indirect indicators cannot fully substitute for direct assessments. To more precisely evaluate the independent effect of phosphate variability, future studies should incorporate more comprehensive and specific clinical and biochemical variables. Furthermore, serum phosphate levels were not standardized across all measurements. Finally, our study investigated all-cause mortality without stratifying by specific causes such as cardiovascular- or infection-related deaths. Due to limitations in the claims database, we could not reliably assess cause-specific mortality. This restricts our ability to evaluate whether phosphate variability differentially affects particular types of mortality. Future studies incorporating detailed clinical or national mortality registry data are warranted to address this important issue.

## 5. Conclusions

**Figure 2:**
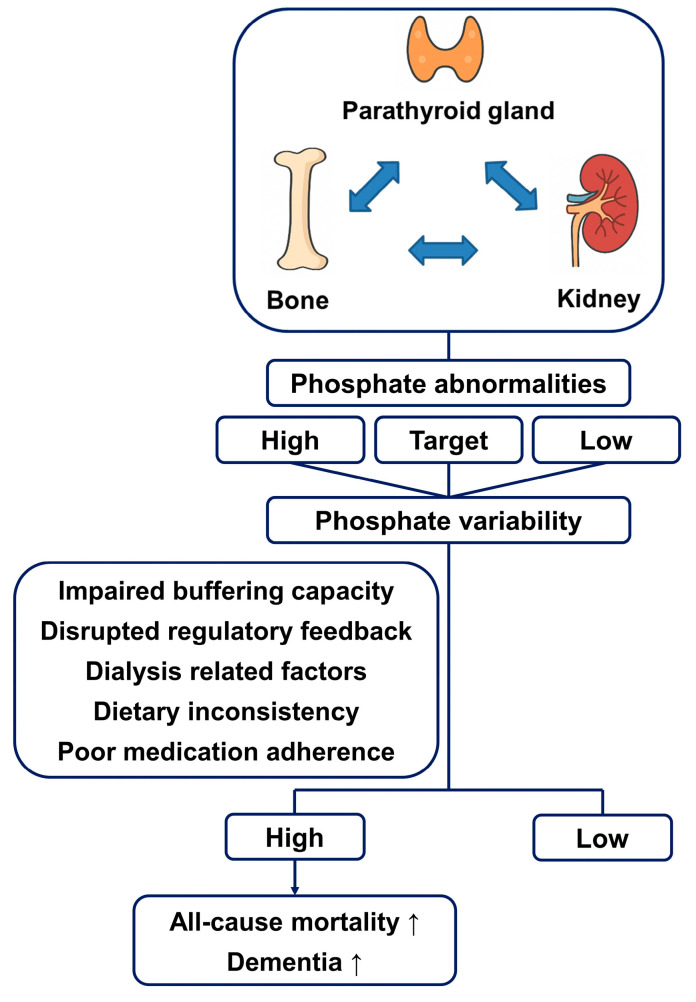
Conceptual diagram illustrating the regulatory interplay between the parathyroid gland, bone, and kidney in phosphate regulation and the implications of phosphate variability.

Our study found an association between high phosphate variability and both all-cause mortality and dementia (Figure 2).

The results observed in most subgroups were consistent with those obtained from the weighted cohort. However, the association between phosphate variability and dementia appeared to be stronger in specific subgroups, including patients with low CCI, female sex, younger age, short HD vintage, or those using PPBs. These findings suggest that phosphate variability may serve as a useful additional indicator for predicting patient prognosis. Nevertheless, due to the inherent limitations of this study, further research is required to confirm this association more definitively.

## Figures and Tables

**Table 1:** Classification of phosphate variability and associated mean phosphate levels in unweighted and weighted cohorts.

	Interval of Residual SD of Phosphate Levels	Unweighted Cohort		Weighted Cohort	
		Mean Phosphate Levels	Residual SDs of Phosphate Levels	Mean Phosphate Levels	Residual SDs of Phosphate Levels
Q1	≤0.47	4.55 ± 1.07	0.34 ± 0.09	4.94 ± 0.01	0.35 ± 0.00
Q2	0.47 < SD ≤ 0.68	4.78 ± 1.05 ^a^	0.57 ± 0.06 ^a^	4.95 ± 0.01	0.57 ± 0.00 ^a^
Q3	0.68 < SD ≤ 0.94	5.08 ± 1.07 ^a,b^	0.80 ± 0.07 ^a,b^	4.99 ± 0.01 ^a,b^	0.79 ± 0.00 ^a,b^
Q4	>0.94	5.47 ± 1.09 ^a,b,c^	1.25 ± 0.29 ^a,b,c^	5.03 ± 0.01 ^a,b,c^	1.22 ± 0.00 ^a,b,c^

Patients were categorized into quartiles based on the residual SD of phosphate levels. Data are presented as mean ± SD for the unweighted cohort and mean ± SE for the weighted cohort. ^a^ *p* < 0.05 vs. Q1; ^b^ *p* < 0.05 vs. Q2; ^c^ *p* < 0.05 vs. Q3. Abbreviations: Q1, first quartile group; Q2, second quartile group; Q3, third quartile group; Q4, fourth quartile group; SD, standard deviation; and SE, standard error.

**Table 2:** Patient clinical characteristics of each cohort using inverse probability of treatment weighting.

	Q1 (*n* = 48,817)	Q2 (*n* = 49,310)	Q3 (*n* = 49,158)	Q4 (*n* = 47,730)	*p*
Age (years)	61.8 ± 0.1	61.9 ± 0.1	61.8 ± 0.1	61.7 ± 0.1	0.612
Sex (male, %)	29,993 (61.4%)	29,987 (60.8%)	29,525 (60.1%)	28,322 (59.3%)	0.011
HD (months)	67 ± 1	67 ± 1	66 ± 1	67 ± 1	0.385
Body mass index (kg/m^2^)	22.8 ± 0.0	22.8 ± 0.0	22.8 ± 0.0	22.7 ± 0.	0.305
Diabetes (%)	21,481 (44.0%)	21,803 (44.2%)	21,701 (44.1%)	20,353 (42.6%)	0.066
CCI score	8.8 ± 0.0	8.8 ± 0.0	8.8 ± 0.0	8.8 ± 0.0	0.079
Arteriovenous fistula (%)	42,385 (86.8%)	42,665 (86.5%)	42,450 (86.4%)	41,046 (86.0%)	0.353
Kt/V_urea_	1.57 ± 0.00	1.57 ± 0.00	1.57 ± 0.00	1.58 ± 0.00	0.016
UFV (L/session)	2.31 ± 0.01	2.30 ± 0.01	2.32 ± 0.01	2.34 ± 0.01	0.001
Hemoglobin (g/dL)	10.7 ± 0.0	10.7 ± 0.0	10.7 ± 0.0	10.7 ± 0.0	0.765
Serum albumin (g/dL)	4.01 ± 0.0	4.01 ± 0.0	4.01 ± 0.0	4.01 ± 0.0	0.865
Serum calcium (mg/dL)	8.9 ± 0.0	8.9 ± 0.0	8.9 ± 0.0	8.9 ± 0.0	0.020
Serum creatinine (mg/dL)	9.5 ± 0.0	9.5 ± 0.0	9.6 ± 0.0	9.7 ± 0.0	<0.001
Use of RASB (%)	32,055 (65.7%)	32,912 (66.7%)	32,757 (66.6%)	31,874 (66.8%)	0.249
Use of aspirin (%)	23,643 (48.4%)	24,326 (49.3%)	23,954 (48.7%)	23,301 (48.8%)	0.606
Use of clopidogrel (%)	12,639 (25.9%)	12,752 (25.9%)	12,561 (25.6%)	12,596 (26.4%)	0.570
Use of statins (%)	23,950 (49.1%)	24,398 (49.5%)	24,720 (50.3%)	23,965 (50.2%)	0.211
Use of anti-HTN drugs (%)	41,440 (84.9%)	41,977 (85.1%)	41,787 (85.0%)	40,634 (85.1%)	0.951
MI or CHF (%)	28,416 (58.2%)	28,571 (57.9%)	28,634 (58.2%)	27,978 (58.6%)	0.795
Atrial fibrillation (%)	5922 (12.1%)	6120 (12.4%)	6154 (12.5%)	5630 (11.8%)	0.375
PPB (%)					<0.001
Calcium-based PPB	30,438 (62.4%)	30,536 (61.9%)	30,717 (62.5%)	29,383 (61.6%)	
Non-calcium-based PPB	13,154 (26.9%)	13,535 (27.4%)	13,706 (27.9%)	14,313 (30.0%)	
No PPB	5225 (10.7%)	5239 (10.6%)	4735 (9.6%)	4034 (8.5%)	
Use of vitamin D agents (%)	26,113 (53.5%)	26,017 (52.8%)	26,198 (53.3%)	25,155 (52.7%)	0.575
Use of cinacalcet (%)	5898 (12.1%)	5950 (12.1%)	5747 (11.7%)	5592 (11.7%)	0.688
Use of CaS (%)	1362 (2.8%)	1499 (3.0%)	1557 (3.2%)	1528 (3.2%)	0.270

Data are expressed as means ± standard error for continuous variables and as *n* (%) for categorical variables. *p*-values were determined using a general linear model with a complex survey design, incorporating sample weights. Abbreviations: anti-HTN, antihypertensive; Q1, first quartile group; Q2, second quartile group; Q3, third quartile group; Q4, fourth quartile group; CaS, calcium supplement; CCI, Charlson comorbidity index; CHF, congestive heart failure; MI, myocardial infarction; PPB, phosphate binder; RASB, renin-angiotensin system blocker; and UFV, ultrafiltration volume.

**Table 3:** HR and 95% CI for clinical outcomes by phosphate variability quartile (weighted cohort).

	Univariable		Multivariable	
	HR (95% CI)	*p*-Value	HR (95% CI)	*p*-Value
All–cause mortality				
Q2	1.03 (1.00–1.05)	0.022	1.03 (1.00–1.05)	0.033
Q3	1.04 (1.01–1.06)	0.002	1.08 (1.05–1.10)	<0.001
Q4	1.06 (1.04–1.09)	<0.001	1.08 (1.06–1.11)	<0.001
Cardiovascular events				
Q2	0.97 (0.93–1.01)	0.111	0.96 (0.93–1.00)	0.085
Q3	1.01 (0.97–1.04)	0.752	1.01 (0.97–1.05)	0.644
Q4	1.01 (0.97–1.05)	0.612	1.02 (0.98–1.07)	0.243
Dementia				
Q2	1.02 (0.97–1.07)	0.526	0.99 (0.94–1.05)	0.782
Q3	1.06 (1.01–1.11)	0.022	1.05 (0.99–1.11)	0.079
Q4	1.12 (1.06–1.18)	<0.001	1.08 (1.02–1.14)	0.005

The multivariate analysis was adjusted for age, sex; body mass index; vascular access type; diabetes status; hemodialysis vintage; Charlson Comorbidity Index score; ultrafiltration volume; Kt/V_urea_; hemoglobin level; serum albumin level; serum creatinine level; mean phosphate level; serum calcium level; use of renin-angiotensin system blockers, statins, clopidogrel, aspirin, antihypertensive drugs, vitamin D agents, phosphate binders, calcium supplements, and cinacalcet; and presence of myocardial infarction or congestive heart failure and atrial fibrillation. Q1 served as the reference group. Abbreviations: Q1–Q4, phosphate variability quartiles; CI, confidence interval; and HR, hazard ratio.

## Data Availability

The raw data were generated at the Health Insurance Review and Assessment Service. The database can be requested from the Health Insurance Review and Assessment Service by sending a study proposal including the purpose of the study, study design, and duration of analysis at the web site (https://www.hira.or.kr accessed on 25 April 2025). The authors cannot distribute the data without permission.

## Supplementary Materials

The following supporting information can be downloaded at: https://www.mdpi.com/article/10.3390/nu17091528/s1, Table S1. Medication types and corresponding Health Insurance Review and Assessment Service codes; Table S2. Patient clinical characteristics of the unweighted cohort; Table S3. HR and 95% CI for all-cause mortality by phosphate variability quartile in the weighted cohort (Q1 as the reference group); Table S4. HR and 95% CI for cardiovascular events by phosphate variability quartile in the weighted cohort (Q1 as the reference group); Table S5. HR for dementia by phosphate variability quartile in the weighted cohort (Q1 as the reference group); Figure S1. Propensity score balance; Figure S2. Spline curves depicting the association between phosphate variability and (A–D) all-cause mortality, (E–H) cardiovascular events, and (I–L) dementia (hazard ratio and 95% confidence interval).
