# Supplementary material for: Impact of Phosphate Variability in Patients Undergoing Hemodialysis
Source: Nutrients. 2025 Apr 30;17(9):1528. doi: 10.3390/nu17091528 (PMC12073743; doi:10.3390/nu17091528)
Supplement: Supplementary file 1 [file nutrients-17-01528-s001.zip › nutrients-3564873-supplementary.pdf]

Supplementary Information

Table S1. Medication types and corresponding Health Insurance Review and Assessment Service codes.

Table S2. Patient clinical characteristics of unweighted cohort.

Table S3. HR and 95% CI for all-cause mortality by phosphate variability quartile in the weighted cohort (Q1 as the reference group).

Table S4. HR and 95% CI for cardiovascular events by phosphate variability quartile in the weighted cohort (Q1 as the reference group).

Table S5. HR and 95% CI for dementia by phosphate variability quartile in the weighted cohort (Q1 as the reference group).

Figure S1. Propensity score balance.

Figure S2. Spline curves depicting the association between phosphate variability and (A–D) all-cause mortality, (E–H) cardiovascular events, and (I–L) dementia.

Table S1. Medication types and corresponding Health Insurance Review and Assessment Service codes.

Medications	Codes
Alacepril	104201ATB, 104202ATB
Benazepril	114701ATB
Captopril	122901ATB, 122902ATB, 122903ATB
Cilazapril	133001ATB, 133002ATB, 133003ATB
Enalapril	151601ATB, 151603ATB
Fosinopril	163501ATB, 163502ATB
Imidapril	173401ATB, 173402ATB
Moexipril	196801ATB, 196802ATB
Lisinopril	184501ATB
Perindopril	211301ATB, 211302ATB, 501601ATB, 501602ATB
Quinapril	221901ATB,
Ramipril	222401ATB, 222402ATB, 222404ATB
Zofenopril	510401ATB, 510402ATB, 510403ATB
Temocapril	235002ATB
Delapril	140901ATB, 140902ATB
Captopril + Hydrochlorothiazide	262200ATB, 262300ATB
Enalapril + Hydrochlorothiazide	440300ATB, 453700ATB, 453600ATB
Ramipril + Felodipine	447100ATB, 447200ATB
Ramipril + Hydrochlorothiazide	448600ATB, 448700ATB
Perindopril + indapamide	556200ATB
Lisinopril + Hydrochlorothiazide	499200ATB, 499300ATB
Moexipril + Hydrochlorothiazide	440800ATB, 497900ATB
Enalapril + Nitrendipine	466000ATB
Candesartan	122601ATB, 122602ATB, 122603ATB
Irbesartan	177301ATB, 177303ATB
Losartan	185701ATB, 185702ATB
Valsartan	247101ATB, 247102ATB, 247103ATB, 247104ATB
Fimasartan	515201ATB, 515202ATB, 515203ATB

Azilsartan	662401ATB, 662402ATB, 662403ATB
Telmisartan	378801ATB, 378802ATB
Eprosartan	429201ATB
Olmesartan	468501ATB, 468502ATB, 468503ATB, 520901ATB, 520902ATB
Valsartan + Amlodipine	492800ATB, 492900ATB, 495800ATB, 522600ABTB, 522700ABTB, 522800ABTB, 522900ABTB, 523000ATB, 523100ATB, 523200ATB, 523300ATB, 523400ATB
Valsartan + Lercanidipne	522200ATB. 522300ATB. 522400ATB
Valsartan + Pitavastatin	634900ATB, 635000ATB, 635100ATB, 635200ATB
Valsartan + Sacubitril	651401ATB, 651402ATB, 651403ATB
Valsartan + Rosuvastatin	629700ATB, 629800ATB, 525000ATB, 525100ATB, 525200ATB, 525300ATB,
Valsartan + Hydrochlorothiazide	356400ATB, 442600ATB
Olmesaetan + Amlodipine	500500ATB, 500600ATB, 547500ATB. 547600ATB, 547700ATB, 547800ATB, 547900ATB, 548000ATB, 582200ATB, 582400ATB, 629400ATB, 629500ATB, 629600ATB, 631300ATB, 632800ATB, 632900ATB, 633000ATB
Olmesartan + Hydrochlorothiazide	513600ATB
Olmesartan + Hydrochlorothiazide + Amlodipine	519700ATB, 519800ATB, 519900ATB, 520000ATB, 520100ATB
Olmesartan + Rosuvastatin	653200ATB, 644100ATB, 644200ATB, 526300ATB, 526400ATB, 526500ATB, 526900ATB
Telmisartan + Hydrochlorothiazide	502600ATB, 443200ATB, 443300ATB
Telmisartan + Rosuvastatin	629900ATB, 630000ATB, 630100ATB, 630200ATB, 631600ATB, 631700ATB
Telmisartan + Amlodipine	511500ATB, 511600ATB, 511700ATB, 521200ATB, 521300ATB, 521400ATB, 623100ATB, 644800ATB
Telmisartan+ Hydrochlorothiazide + Amlodipine	663500ATB, 663600ATB, 663700ATB, 663800ATB
Telmisartan + Rosuvastatin + Amlodipine	671700ATB, 671600ATB, 671500ATB, 671400ATB, 671300ATB, 671200ATB,
Losartan + Hydrochlorothiazide	262500ATB, 378900ATB, 486900ATB
Losartan + Amlodipine	502700ATB, 503000ATB, 513900ATB, 637400ATB, 637500ATB, 637600ATB
Losartan+Rosuvastatin+ Amlodipine	663900ATB, 664000ATB, 664100ATB, 664200ATB, 664300ATB, 664400ATB,
Losartan+Hydrochlorothiazide+ Amlodipine	662800ATB, 662900ATB, 663000ATB
Fimasartan + Hydrochlorothiazide	522000ATB, 526800ATB
Fimasartan + Amlodipine	651900ATB, 652000ATB, 652100ATB, 652700ATB, 651900ATB
Fimasartan + Rosuvastatin	654600ATB, 654700ATB, 654800ATB, 654900ATB, 655000ATB

Candesartan + Hydrochlorothiazide	423700ATB
Candesartan + Amlodipine	652900ATB, 653000ATB, 653100ATB, 652900ATB, 652900ATB
Candesartan + Rosuvastatin	673700ATB, 661800ATB, 661900ATB, 662000ATB, 662100ATB
Irbesartan + Hydrochlorothiazide	385700ATB, 385800ATB
Irbesartan + Atorvastatin	527000ATB, 527100ATB, 524000ATB, 524100ATB
Azilsartan + Chlorthalidone	673500ATB, 673600ATB
Eprosartan + Hydrochlorothiazide	460500ATB
Amlodipine + Atorvastatin	614500ATB, 472300ATB, 472400ATB, 472500ATB, 518900ATB
Amlodipine + Rosuvastatin	673900ATB, 674000ATB, 674100ATB
Atorvastatin + Ezetimibe	633800ATB, 633900ATB, 634800ATB
Pitavastatin + Fenofibrate	679300ACH
Rosuvastatin + Ezetimibe	640700ATB, 640800ATB, 640900ATB
Metformin + Atorvastatin	671800ATR, 673800ATR, 671900ATR, 672000ATR, 672100ATR
Metformin + Rosuvastatin	672500ATR, 672600ATR, 672700ATR, 672800ATR, 672900ATR, 673000ATR, 683300ATR, 683400ATR
Gemigliptin + Rosuvastatin	664600ATB, 664700ATB, 664800ATB
Aspirin	110701ATB, 110702ATB, 110801ATB, 110802ATB, 111001ACE, 111001ATB, 111001ATE, 111002ATE, 111003ACE, 111003ATE
Clopidogrel	133201ACR, 133201ATB, 133201ATR, 133202ATB, 133203ATR, 506100ATB
Cilostazol	136901ATB, 492501ATB, 495201ATB, 498801ATB, 501501ATB
Ticlopidine	498900ATB, 239201ATB, 239202ATB
Aspirin + Bethocarbamol	256800ATB
Aspirin + Clopidogrel	517900ACH, 517900ACE, 517900ATE, 667500ACE
Aspirin + Dipyridamole	489700ACR
Atorvastatin	111502ATB, 502202ATB, 633900ATB, 472400ATB, 518900ATB, 524100ATB, 527000ATB, 672000ATR, 672100ATR, 111503ATB, 502203ATB, 634800ATB, 472500ATB, 111504ATB, 502204ATB
Fluvastatin	162401ACH, 162402ACH, 162403ATR

Lovastatin	185801ATB
Pitavastatin	470901ATB, 470902ATB, 470903ATB
Pravastatin	216601ATB, 216602ATB, 216603ATB, 216604ATB
Rosuvastatin	454001ATB, 454002ATD, 454002ATB, 454003ATB, 454003ATD, 454005ATB
Simvastatin	227801ATB, 227802ATB, 227803ATB, 227805ATB, 227806ATB
Calcium carbonate	121801ATB, 121901ATB,
Calcium acetate	121701ATB
Calcium citrate	122101ATB
Paricalcitol	430703BIJ, 430702BIJ, 430701BIJ, 430730BIJ, 430731BIJ
Alfacalcidol	104601ACS, 104601ATB, 104602ACS
Calcitriol	121601ACS, 121602BIJ, 121630BIJ
Calcifediol	121401ACS, 121402ACS
Cinacalcet	512301ATB, 512302ATB
Cholecalciferol + Calcium carbonate	302600ATB, 387900ACS, 409100ATB, 473800ATB, 480200ATB, 498200ATB, 498300ATB, 526100ATB, 634000ATB, 521900ATB
Cholecalciferol + Calcium citrate	462700ATB, 462800ATB, 519000ATB, 503500ATB, 504400ATB, 508700ATB, 665600ATB, 670000ATB
Cholecalciferol + Calcium citrate	503100ATB
Ergocalciferol + Calcium gluconate+ Calcium phosphate	473300ACS
Ergocalciferol + Calcium carbonate+ Calcium gluconate + Calcium lactate	303200ATB
Cholecalciferol + Alendronate	481100ATB, 500200ATB
Cholecalciferol + Ibandronate	523900ATB
Cholecalciferol + Risendronate	511200ATB, 518400ATB
Calcitriol + Alendronate	468000ATE
Cholecalciferol + Ralxifene	659200ACH, 659200ATB
Cholecalciferol + Bazedoxifene	674500ATB
Sevelamer	428501ATB, 428502ATB, 517701APD, 517701ATB
Lanthanum	487101ATB, 487102ATB, 487103ATB, 487104ATB

Table S2. Patient clinical characteristics of unweighted cohort.

	Q1 (n = 12,557)	Q2 (n = 12,556)	Q3 (n = 12,556)	Q4 (n = 12,556)	*P*
Age (years)	64.1 ± 12.7	62.7 ± 12.6[a]	61.3 ± 12.7[a,b]	59.7 ± 12.8[a,b,c]	<0.001
Sex (male, %)	7,902 (62.9%)	7,556 (60.2%)	7,588 (60.4%)	7,401 (58.9%)	<0.001
HD (months)	65 ± 69	66 ± 68	67 ± 68[a]	68 ± 67[a]	<0.001
Body mass index (kg/m^2)	22.7 ± 3.5	22.7 ± 3.5	22.8 ± 3.6[a]	22.8 ± 3.6[a]	0.013
Diabetes (%)	5,702 (45.4%)	5,657 (45.1%)	5,485 (43.7%)	5,188 (41.3%)	<0.001
CCI score	8.9 ± 2.9	8.8 ± 2.8[a]	8.8 ± 2.9[a]	8.8 ± 2.8[a]	<0.001
Arteriovenous fistula (%)	10,742 (85.5%)	10,809 (86.1%)	10,880 (86.7%)	10,906 (86.9%)	0.011
Kt/V_{urea}	1.56 ± 0.27	1.57 ± 0.27	1.57 ± 0.28	1.57 ± 0.28	0.079
UFV (L/session)	2.16 ± 0.91	2.25 ± 0.87[a]	2.36 ± 0.85[a,b]	2.45 ± 0.83[a,b,c]	<0.001
Hemoglobin (g/dL)	10.7 ± 0.7	10.6 ± 0.7[a]	10.6 ± 0.7[a]	10.7 ± 0.7	<0.001
Serum albumin (g/dL)	3.98 ± 0.33	4.00 ± 0.33[a]	4.02 ± 0.33[a,b]	4.04 ± 0.32[a,b,c]	<0.001
Serum calcium (mg/dL)	8.8 ± 0.7	8.9 ± 0.7[a]	8.9 ± 0.7[a,b]	8.9 ± 0.7[a,b]	<0.001
Serum creatinine (mg/dL)	8.8 ± 2.7	9.3 ± 2.6[a]	9.8 ± 2.5[a,b]	10.3 ± 2.5[a,b,c]	<0.001
Use of RASB (%)	7,996 (63.7%)	8,344 (66.5%)	8,400 (66.9%)	8,515 (67.8%)	<0.001
Use of aspirin (%)	6,106 (48.6%)	6,183 (49.2%)	6,108 (48.6%)	6,099 (48.6%)	0.682
Use of clopidogrel (%)	3,429 (27.3%)	3,272 (26.1%)	3,157 (25.1%)	3,220 (25.6%)	<0.001
Use of statins (%)	6,345 (50.5%)	6,271 (49.9%)	6,277 (50.0%)	6,172 (49.2%)	0.185
Use of anti-HTN drugs (%)	10,513 (83.7%)	10,668 (85.0%)	10,693 (85.2%)	10,783 (85.9%)	<0.001
MI or CHF (%)	7,359 (58.6%)	7,276 (57.9%)	7,277 (58.0%)	7,419 (59.1%)	0.193
Atrial fibrillation (%)	1,628 (13.0%)	1,582 (12.6%)	1,564 (12.5%)	1,391 (11.1%)	<0.001
PPB (%)					<0.001
Calcium-based PPB	7,650 (60.9%)	7,947 (63.3%)	7,971 (63.5%)	7,475 (59.5%)	
Non-calcium-based PPB	2,545 (20.3%)	3,116 (24.8%)	3,681 (29.3%)	4,630 (36.9%)	
No PPB	2,362 (18.8%)	1,493 (11.9%)	904 (7.2%)	451 (3.6%)	
Use of vitamin D agents (%)	6,302 (50.2%)	6,488 (51.7%)	6,824 (54.3%)	6,917 (55.1%)	<0.001
Use of cinacalcet (%)	1,219 (9.7%)	1,412 (11.2%)	1,555 (12.4%)	1,699 (13.5%)	<0.001
Use of CaS (%)	379 (3.0%)	402 (3.2%)	380 (3.0%)	389 (3.1%)	0.823

Data are expressed as means ± standard deviation for continuous variables and as n (%) for categorical variables. *P*-values were calculated using a one-way analysis of variance (ANOVA) with Tukey's post hoc test (continuous variables) and Pearson's χ^2 test (categorical variables). [a]*P* < 0.05 vs. Q1, [b]*P* < 0.05 vs. Q2, [c]*P* < 0.05 vs. Q3.

Abbreviations: anti-HTN, antihypertensive; Q1, first quartile group; Q2, second quartile group; Q3, third quartile group; Q4, fourth quartile group; CaS, calcium supplement; CCI, Charlson comorbidity index; CHF, congestive heart failure; MI, myocardial infarction; PPB, phosphate binder; RASB, renin-angiotensin system blocker; UFV, ultrafiltration volume.

Table S3. HR and 95% CI for all-cause mortality by phosphate variability quartile in the weighted cohort (Q1 as the reference group).

	Multivariate			Multivariate	
	HR (95% CI)	*P*		HR (95% CI)	*P*
**Males**			**Females**		
Q2	1.02 (0.99–1.06)	0.139		1.04 (1.00–1.09)	0.044
Q3	1.06 (1.02–1.09)	<0.001		1.12 (1.07–1.16)	<0.001
Q4	1.10 (1.07–1.14)	<0.001		1.05 (1.01–1.10)	0.012
**<65 years old**			**≥65**		
Q2	1.00 (0.96–1.04)	0.974		1.04 (1.01–1.07)	0.010
Q3	1.02 (0.98–1.07)	0.339		1.10 (1.07–1.13)	<0.001
Q4	1.15 (1.10–1.20)	<0.001		1.04 (1.01–1.08)	0.005
**CCI < 8**			**CCI ≥ 8**		
Q2	0.95 (0.90–1.00)	0.072		1.05 (1.02–1.08)	<0.001
Q3	0.94 (0.89–0.99)	0.025		1.11 (1.08–1.14)	<0.001
Q4	1.06 (1.00–1.12)	0.040		1.09 (1.06–1.12)	<0.001
**HDV < 41 months**			**HDV ≥ 41**		
Q2	0.99 (0.95–1.03)	0.604		1.06 (1.02–1.09)	<0.001
Q3	1.09 (1.05–1.13)	<0.001		1.07 (1.03–1.10)	<0.001
Q4	1.14 (1.10–1.18)	<0.001		1.02 (0.99–1.06)	0.166
**CPPB**			**NCPPB**		
Q2	1.02 (0.99–1.05)	0.199		1.03 (1.00–1.05)	0.033
Q3	1.07 (1.04–1.10)	<0.001		1.08 (1.05–1.10)	<0.001
Q4	1.11 (1.08–1.15)	<0.001		1.08 (1.06–1.11)	<0.001
**No PPB**					
Q2	1.03 (0.96–1.09)	0.419			
Q3	1.10 (1.03–1.17)	0.003			
Q4	0.92 (0.86–0.99)	0.021			
**Phos < 3.5mg/dL**			**3.5 ≤ Phos ≤ 5.5**		
Q2	0.98 (0.91–1.05)	0.531		1.05 (1.02–1.09)	<0.001
Q3	1.19 (1.11–1.28)	<0.001		1.08 (1.04–1.11)	<0.001
Q4	0.74 (0.68–0.81)	<0.001		1.14 (1.11–1.18)	<0.001
**Phos > 5.5**					
Q2	0.97 (0.92–1.03)	0.329			
Q3	1.02 (0.96–1.07)	0.520			

Q4	1.04 (0.99–1.10)	0.108

The multivariate analysis was adjusted for age, sex, body mass index, vascular access type, diabetes, hemodialysis vintage, CCI score, ultrafiltration volume, Kt/V_{urea}, hemoglobin level, serum albumin level, serum creatinine level, mean phosphorus level, serum calcium level; use of renin-angiotensin system blockers, statins, clopidogrel, aspirin, antihypertensive drugs, vitamin D agents, phosphate binders, calcium supplements, and cinacalcet; and presence of myocardial infarction or congestive heart failure, and atrial fibrillation.

Abbreviations: CCI, Charlson Comorbidity Index; CI, confidence interval; HDV, hemodialysis vintage; HR, hazard ratio; CPPB, calcium-based phosphate binder; NCPPB, non-calcium based phosphate binder; PPB, phosphate binder; Phos, mean phosphorus level.

Table S4. HR and 95% CI for cardiovascular events by phosphate variability quartile in the weighted cohort (Q1 as the reference group).

	Multivariate			Multivariate	
	HR (95% CI)	*P*		HR (95% CI)	*P*
**Males**			**Females**		
Q2	0.91 (0.87–0.96)	<0.001		1.06 (0.99–1.13)	0.102
Q3	0.98 (0.93–1.03)	0.377		1.07 (0.99–1.14)	0.052
Q4	1.00 (0.95–1.05)	0.966		1.07 (0.99–1.14)	0.054
**< 65 years old**			**≥ 65**		
Q2	0.99 (0.94–1.05)	0.766		0.94 (0.88–0.99)	0.026
Q3	1.01 (0.95–1.07)	0.780		1.00 (0.95–1.06)	0.929
Q4	1.03 (0.97–1.09)	0.383		1.01 (0.96–1.08)	0.628
**CCI < 8**			**CCI ≥ 8**		
Q2	0.95 (0.88–1.02)	0.155		0.97 (0.93–1.02)	0.274
Q3	0.96 (0.89–1.03)	0.292		1.03 (0.98–1.08)	0.203
Q4	1.00 (0.93–1.07)	0.926		1.04 (0.99–1.09)	0.165
**HDV < 41 months**			**HDV ≥ 41**		
Q2	1.02 (0.96–1.08)	0.509		0.92 (0.87–0.97)	0.002
Q3	1.01 (0.95–1.07)	0.700		1.01 (0.96–1.07)	0.717
Q4	1.00 (0.94–1.07)	0.895		1.04 (0.99–1.10)	0.141
**CPPB**			**NCPPB**		
Q2	0.95 (0.90–0.99)	0.035		0.96 (0.93–1.00)	0.085
Q3	0.98 (0.93–1.03)	0.357		1.01 (0.97–1.05)	0.644
Q4	1.02 (0.97–1.07)	0.414		1.02 (0.98–1.07)	0.243
**No PPB**					
Q2	1.08 (0.95–1.22)	0.239			
Q3	1.27 (1.12–1.44)	<0.001			
Q4	0.96 (0.84–1.11)	0.608			
**Phos < 3.5mg/dL**			**3.5 ≤ Phos ≤ 5.5**		
Q2	1.02 (0.89–1.18)	0.760		0.95 (0.90–0.99)	0.047
Q3	0.94 (0.81–1.09)	0.386		1.03 (0.98–1.08)	0.259
Q4	0.88 (0.73–1.05)	0.164		1.06 (1.01–1.12)	0.021
**Phos > 5.5**					
Q2	0.98 (0.91–1.06)	0.700			
Q3	0.97 (0.90–1.05)	0.430			

Q4	0.98 (0.91–1.06)	0.668

The multivariate analysis was adjusted for age, sex, body mass index, vascular access type, diabetes, hemodialysis vintage, CCI score, ultrafiltration volume, Kt/V_{urea}, hemoglobin level, serum albumin level, serum creatinine level, mean phosphorus level, serum calcium level; use of renin-angiotensin system blockers, statins, clopidogrel, aspirin, antihypertensive drugs, vitamin D agents, phosphate binders, calcium supplements, and cinacalcet; and presence of myocardial infarction or congestive heart failure, and atrial fibrillation.

Abbreviations: CCI, Charlson Comorbidity Index; CI, confidence interval; HDV, hemodialysis vintage; HR, hazard ratio; CPPB, calcium-based phosphate binder; NCPPB, non-calcium based phosphate binder; PPB, phosphate binder; Phos, mean phosphorus level.

Table S5. HR and 95% CI for dementia by phosphate variability quartile in the weighted cohort (Q1 as the reference group).

	Multivariate			Multivariate	
	HR (95% CI)	*P*		HR (95% CI)	*P*
**Males**			**Females**		
Q2	1.03 (0.96–1.11)	0.362		0.93 (0.86–1.01)	0.090
Q3	0.96 (0.89–1.03)	0.282		1.16 (1.07–1.25)	<0.001
Q4	1.02 (0.95–1.10)	0.571		1.15 (1.06–1.25)	<0.001
**< 65 years old**			**≥ 65**		
Q2	0.86 (0.79–0.95)	0.002		1.07 (0.99–1.14)	0.052
Q3	0.92 (0.84–1.01)	0.087		1.12 (1.04–1.19)	0.001
Q4	1.14 (1.05–1.25)	0.003		1.04 (0.97–1.11)	0.308
**CCI < 8**			**CCI ≥ 8**		
Q2	0.93 (0.83–1.05)	0.225		1.01 (0.95–1.08)	0.707
Q3	1.13 (1.01–1.26)	0.034		1.03 (0.97–1.09)	0.358
Q4	1.21 (1.09–1.36)	<0.001		1.04 (0.98–1.11)	0.179
**HDV < 41 months**			**HDV ≥ 41**		
Q2	1.04 (0.96–1.12)	0.324		0.95 (0.88–1.02)	0.182
Q3	1.06 (0.98–1.14)	0.158		1.04 (0.97–1.12)	0.303
Q4	1.09 (1.01–1.18)	0.036		1.07 (0.99–1.15)	0.092
**CPPB**			**NCPPB**		
Q2	0.95 (0.89–1.02)	0.135		0.99 (0.94–1.05)	0.782
Q3	1.02 (0.96–1.09)	0.480		1.05 (0.99–1.11)	0.079
Q4	1.12 (1.05–1.20)	<0.001		1.08 (1.02–1.14)	0.005
**No PPB**					
Q2	1.19 (1.03–1.39)	0.021			
Q3	1.46 (1.25–1.70)	<0.001			
Q4	0.86 (0.72–1.03)	0.103			
**Phos < 3.5mg/dL**			**3.5 ≤ Phos ≤ 5.5**		
Q2	1.12 (0.95–1.32)	0.182		0.95 (0.89–1.02)	0.160
Q3	1.22 (1.03–1.45)	0.024		1.03 (0.97–1.10)	0.364
Q4	0.67 (0.54–0.83)	<0.001		1.09 (1.02–1.16)	0.009
**Phos > 5.5**					
Q2	1.04 (0.92–1.17)	0.541			
Q3	1.03 (0.91–1.16)	0.640			

Q4	1.18 (1.06–1.33)	0.004

The multivariate analysis was adjusted for age, sex, body mass index, vascular access type, diabetes, hemodialysis vintage, CCI score, ultrafiltration volume, Kt/V_{urea}, hemoglobin level, serum albumin level, serum creatinine level, mean phosphorus level, serum calcium level; use of renin-angiotensin system blockers, statins, clopidogrel, aspirin, antihypertensive drugs, vitamin D agents, phosphate binders, calcium supplements, and cinacalcet; and presence of myocardial infarction or congestive heart failure, and atrial fibrillation.

Abbreviations: CCI, Charlson Comorbidity Index; CI, confidence interval; HDV, hemodialysis vintage; HR, hazard ratio; CPPB, calcium-based phosphate binder; NCPPB, non-calcium based phosphate binder; PPB, phosphate binder; Phos, mean phosphorus level.

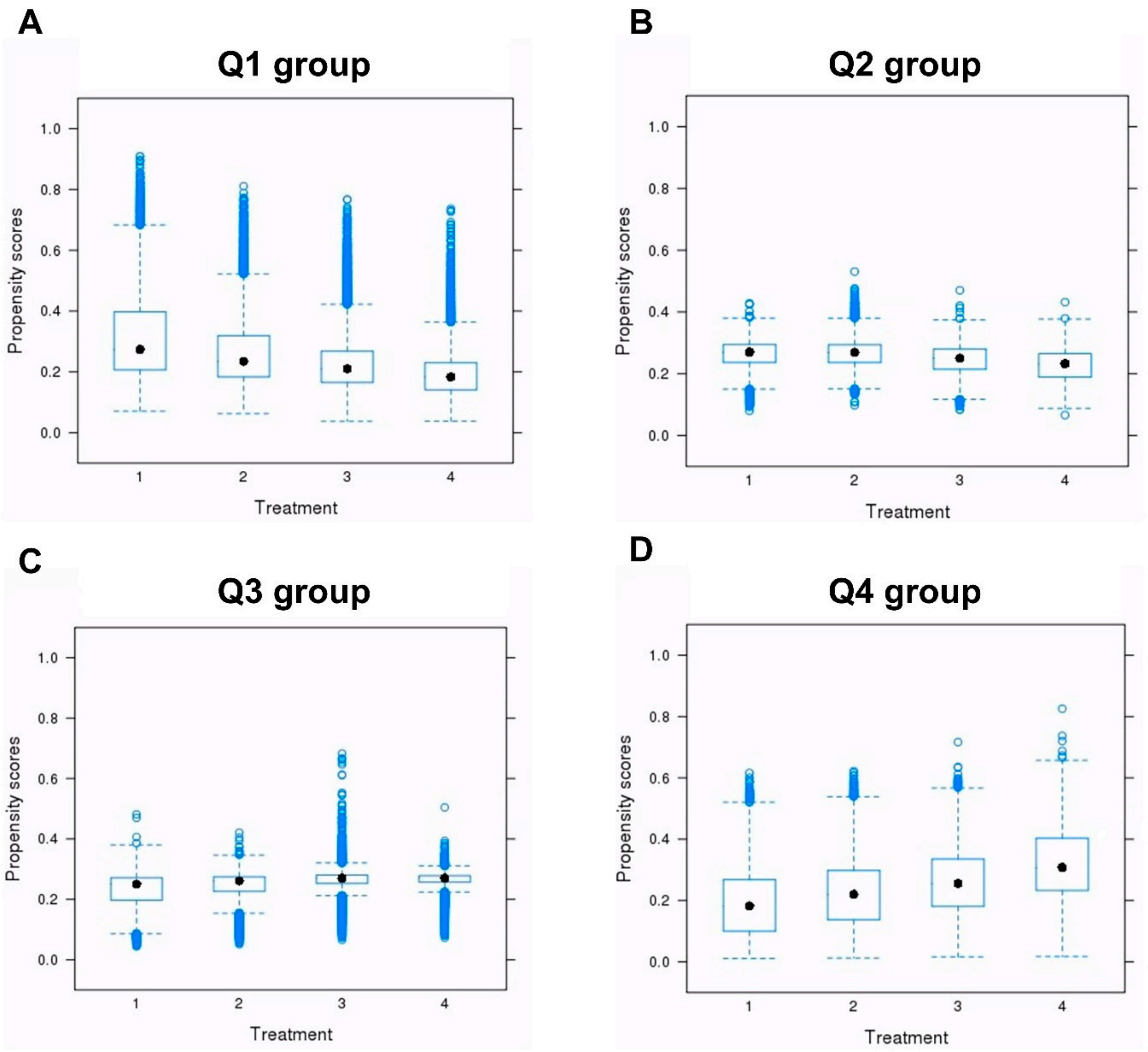

Figure S1. Propensity score balance. (A–D) Box plots showing the distribution of propensity scores for each phosphate variability quartile (Q1, Q2, Q3, and Q4). Median propensity scores within each quartile are indicated by filled black circles.

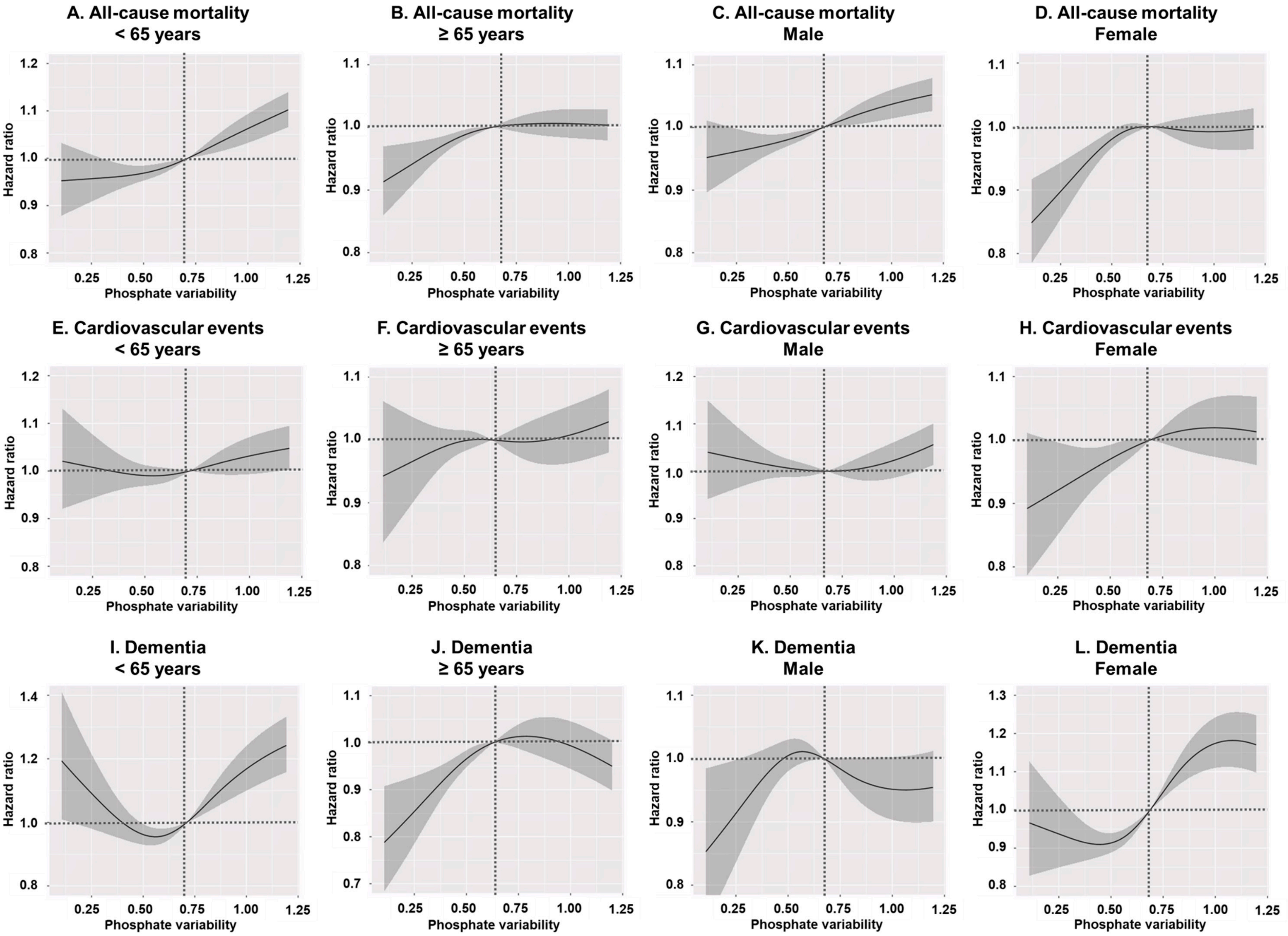

A. All-cause mortality
< 65 years
B. All-cause mortality
≥ 65 years
C. All-cause mortality
Male
D. All-cause mortality
Female
E. Cardiovascular events
< 65 years
F. Cardiovascular events
≥ 65 years
G. Cardiovascular events
Male
H. Cardiovascular events
Female
I. Dementia
< 65 years
J. Dementia
≥ 65 years
K. Dementia
Male
L. Dementia
Female
Hazard ratio
Phosphate variability
0.25
0.50
0.75
1.00
1.25
0.7
0.8
0.9
1.0
1.1
1.2
1.3
1.4

Figure S2. Spline curves depicting the association between phosphate variability and (A–D) all-cause mortality, (E–H) cardiovascular events, and (I–L) dementia (hazard ratio and 95% confidence interval). Panels A, E, and I: patients aged <65 years. Panels B, F, and J: patients aged ≥ 65 years. Panels C, G, and K: male patients. Panels D, H, and L: female patients. Curves are adjusted for age, sex, body mass index, vascular access type, diabetes status, hemodialysis vintage, Charlson Comorbidity Index, ultrafiltration volume, Kt/V_{urea}, hemoglobin, serum albumin, serum creatinine, mean phosphate and serum calcium levels, use of renin-angiotensin system blockers, statins, clopidogrel, aspirin, antihypertensive medications, vitamin D analogs, phosphate binders, calcium supplements, cinacalcet, and presence of myocardial infarction, congestive heart failure, or atrial fibrillation. Dot line reveal 1.0 value of hazard ratio for patients with 0.68 mg/dL of phosphate variability.
